# Identifying relationships between unrelated pharmaceutical target proteins on the basis of shared active compounds

**DOI:** 10.4155/fsoa-2017-0037

**Published:** 2017-06-01

**Authors:** Filip Miljković, Ryo Kunimoto, Jürgen Bajorath

**Affiliations:** 1 Department of Life Science Informatics, B-IT, LIMES Program Unit Chemical Biology & Medicinal Chemistry, Rheinische Friedrich-Wilhelms-Universität, Dahlmannstr. 2, D-53113 Bonn, Germany

**Keywords:** bioactive compounds, compound-based target relationships, ligand-binding characteristics, target families, target proteins

## Abstract

**Aim:**

Computational exploration of small-molecule-based relationships between target proteins from different families.

**Materials & methods:**

Target annotations of drugs and other bioactive compounds were systematically analyzed on the basis of high-confidence activity data.

**Results:**

A total of 286 novel chemical links were established between distantly related or unrelated target proteins. These relationships involved a total of 1859 bioactive compounds including 147 drugs and 141 targets.

**Conclusion:**

Computational analysis of large amounts of compounds and activity data has revealed unexpected relationships between diverse target proteins on the basis of compounds they share. These relationships are relevant for drug discovery efforts. Target pairs that we have identified and associated compound information are made freely available.

## Lay abstract

Relationships between proteins are usually studied by comparing their sequences and functions. However, in addition to biological relationships, chemical links between proteins can also be established by searching for active compounds they share. If proteins have active compounds in common, they are likely to interact with small molecules in similar ways, which provides important clues for drug discovery. Therefore, we have systematically searched for unexpected compound-based relationships between proteins.

## Graphical abstract

Shown here are exemplary small molecules that are active against two targets with different functions. Thus, these compounds establish an unexpected chemical/ligand-binding relationship between these targets.

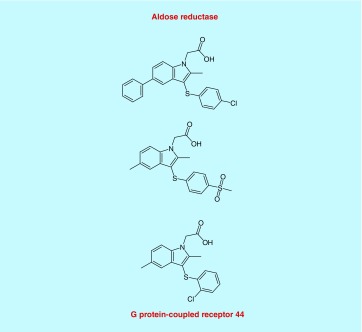

Relationships between proteins are typically derived on the basis of evolutionary criteria including sequence similarity and functions [1–5]. This also applies to the organization of protein families and the assignment of new proteins to existing families [4,5]. In addition to biological considerations, relationships between proteins can also be established on the basis of ligands they share [6–9]. This approach especially applies to pharmaceutically relevant targets capable of interacting with small molecules [10,11]. If such targets share active compounds, they are likely to have similar binding characteristics. These chemical relationships are relevant for drug discovery. A variety of attempts have been made to systematically account for currently available protein–small molecule interactions [12–21]. Many of these investigations have focused on target prediction for drugs or other bioactive compounds. As a representative example, Nicola *et al.* [20] have implemented workflows to arrive at knowledge-supported target hypotheses for active compounds taking chemical similarity into account.

The idea to establish relationships between target proteins on the basis of active compounds originated about a decade ago when the notion of polypharmacology emerged [6]. Evidence was accumulating that many drugs and other bioactive compounds interacted with multiple rather than single targets [6]. Multitarget activities were found to be responsible for desired pharmacological effects, but also for undesired site effects [7]. Such insights also gave rise to more global views of target–compound interactions, leading to the design of network representations linking chemical and target space [6–8]. Clearly, drugs and other compounds with unexpected multitarget activities were prime candidates to further explore questions relating to drug efficacy and pharmacology. Moreover, if target proteins shared active compounds, indicating similar ligand-binding properties, a new dimension would be added to biological relationships and implications of such chemical links for drug discovery could be explored.

Compared with the situation a decade ago, compound activity data have grown in an unforeseeable manner [22–25]. In the public domain, millions of compounds have become available that are active against more than 10,000 targets [23,25]. These are astonishing numbers that would have been hard to imagine just a few years ago. Recent growth in compounds and activity data thus provides unprecedented opportunities to, for example, further explore molecular origins of polypharmacology [23] or investigate compound-based target relationships. This has prompted us to conduct a systematic search for small molecule connections between distantly related or unrelated targets, as reported in the following. Our analysis does not involve predictions of targets for given drugs or other active compounds. Rather, it is based upon a systematic data mining effort to analyze target annotations of small molecules and establish compound-based target pairs.

## Materials & methods

### Compounds & activity data

Bioactive compounds were assembled from ChEMBL version 22 [25]. ChEMBL compounds originate from medicinal chemistry publications and are often (but not always) the result of chemical optimization efforts. Only compounds involved in direct interactions (target relationship type ‘D’) with human targets at the highest confidence level (target confidence score 9) were selected. As potency measurements, only numerically defined equilibrium constants (K_i_ values) and IC_50_ values were considered. Approximate measurements indicated by ‘>,’ ‘<,’ or ‘∼’ were discarded. If a compound had multiple K_i_ or IC_50_ values for the same target, the geometric mean of the values was calculated as the final potency annotation, provided all values fell into the same order of magnitude. Otherwise, the values were disregarded. Because only high-confidence activity data were considered in our analysis and weak compound activities were worth considering for establishing remote target relationships, no potency cutoff was applied. Furthermore, we note that records of inactivity in different assays are not available in ChEMBL. Compound structures were standardized using the OpenEye OEChem toolkit [26]. Standardization involved removal of salts and solvent molecules from compound records, conversion of isotopes, removal of stereoisomers, neutralization of bases and acids and canonicalization of the representation of aromatic structures.

Applying these selection criteria focusing on high-confidence activity data, a total of 224,532 unique compounds were obtained with activity against human 1687 targets. Antitargets belonging to the hERG and CYP450 families and compounds active against them were not considered. By definition, compounds used to establish target relationships must have at dual- or multitarget activity. However, prior to searching for compound-based target relationships, preselected compounds were filtered for potential assay interference compounds [25,27] and aggregators [28] to flag those that may give rise to artifacts (despite the exclusive use of high-confidence activity data), leading to the removal of a total of 10,606 questionable compounds. Application of these filters, which cannot be expected to be perfect, might also remove compounds with true activity. On the other hand, other compounds with interference characteristics might exist. However, removal of more than 10,000 potential candidates for assay interference represented a conservative approach in the context of our study.

### Target assignments

Two target assignment protocols were applied in parallel. First, targets were selected that could be assigned to specific families on the basis of the UniProt classification scheme [29], referred to as ‘family-based’ selection. Second, target pairs were enumerated and pairs with similar sequences (identity > 20% or at least 30 identical residues) were removed [30], referred to as ‘sequence-based’ selection. For selected targets, additional information was retrieved from the Therapeutic Target Database [31] and from KEGG [32].

### Compound-based target pairs

For the formation of a target pair, we required at least three available active compounds. For family-based selection, all pairs of targets were determined that originated from different families (pairs originating from the same family were not considered). For sequence-based selection, all pairs of sequence-diverse targets were identified which shared active compounds. Then, the intersection of the two sets of compound-based target pairs was formed. Approved drugs involved in forming these target pairs were identified using DrugBank [33].

### Network representation

For selected pairs, a compound-based target network was generated using Gephi [34] in which targets were nodes that were connected if they shared active compounds. The network layout was generated using the force-directed Fruchterman–Reingold algorithm [35].

## Results & discussion

### Compound-based target relationships

**Figure 1.**
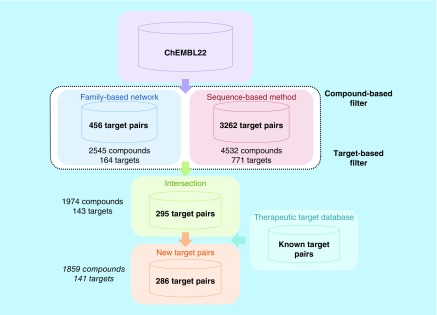
**Identification of compound-based target pairs.** A summary of the analysis is presented, as described in the text.

We have systematically searched for pairs of distantly related or unrelated targets sharing active compounds on the basis of high-confidence activity data. A summary is shown in Figure 1. For targets belonging to different families, 456 compound-based target pairs were identified that involved 2545 unique active compounds and 164 unique targets. In addition, for sequence-diverse targets, 3262 pairs were identified involving 4532 compounds and 771 targets. Determining the intersection of these two sets focused on the analysis of relationships between targets that belonged to different families and had low sequence identity, in other words, distantly or unrelated targets, and yielded 295 target pairs with 1974 compounds and 143 targets. Database searches revealed that only nine of these target relationships were previously reported, leading to our final set of 286 new target pairs that were based upon a total of 1859 active compounds and comprised 141 different targets.’ The compounds involved in these relationships included 147 drugs [33].

### Compound frequency

**Figure 2.**
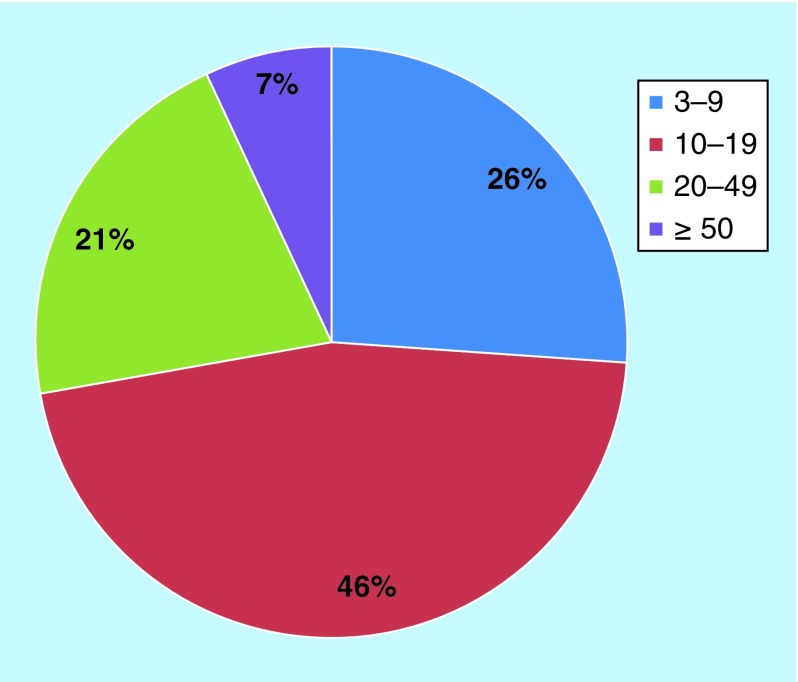
**Compound frequency.** The pie chart reports the percentages of target pairs that were formed by increasing numbers of active compounds (i.e., 3–9, 10–19, 20–49 or at least 50 compounds).

How strong were the chemical links between different targets? In other words, how many active compounds participated in the formation of target pairs? In Figure 2, the distribution of compounds over target pairs is provided. We determined that 21% of the pairs shared 3–9 active compounds; 46%, 10–19; 21%, 20–49; and 7% of the pairs at least 50 compounds. Thus, target pairs were frequently established on the basis of large numbers of compounds, lending further credence to unexpected chemical relationships between different targets.

### Targets

**Figure 3.**
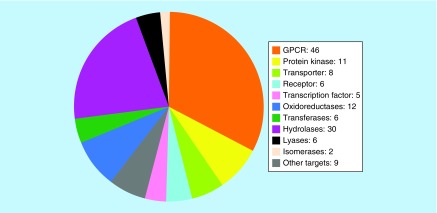
**Target distribution.** The distribution of targets from pairs over different families is shown.

Figure 3 shows the distribution of different targets over families for which compound-based relationships were established. Dominant among these targets were G-protein coupled receptors (GPCRs, 46 members) and protein kinases with 11 members. GPCRs and kinases currently represent most popular pharmaceutical targets. In addition, targets from pairs included a variety of enzymes including hydrolases (30 members) and oxidoreductases (12) as well as different transporter proteins (8), transcription factors (5) and other (non-GPCR) receptors (6). Thus, distantly related or unrelated targets forming pairs originated from diverse families having different functions.

### Composition of target pairs

**Figure 4.**
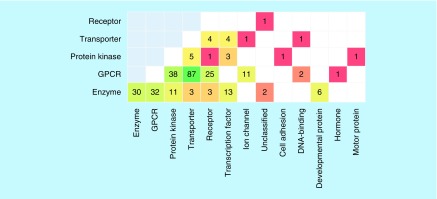
**Target pair composition.** The distribution of target pairs over different combinations of families is shown. Matrix cells representing family combinations and are colored by population using a color spectrum from red (one pair) over yellow to dark green (largest number of pairs).

Figure 4 shows families from which targets forming compound-based relationships originated. Many different – and unexpected – combinations of families were observed; some of which were frequent, whereas others were not. For example, interesting unique combinations included, among others, a cell adhesion protein and a kinase or a DNA-binding protein and a transporter. More frequent combinations included, for example, 13 instances of transcription factors and various enzymes. The most frequent combinations involved GPCRs and different enzymes (32 examples), kinases and GPCRs (38) as well as transporters and GPCRs (87), by far the most frequent pairing.

**Figure 5.**
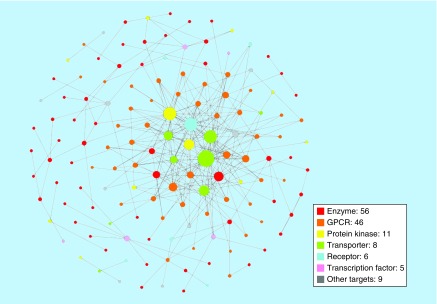
**Compound-based target network.** For targets comprising 286 new pairs, a compound-based target network is shown. Nodes represent targets that are connected by an edge if they share active compounds. In addition, nodes are color-coded by target family (different enzyme families according to Figure 3 are combined into one family; red) and scaled in size by their degree (i.e., number of relationships with other targets).

Were there dominating targets participating in many relationships? To address this question, a compound-based target network was generated (Figure 5). Network analysis revealed that only a few targets were involved in many different relationships, notably small numbers of transporter proteins and kinases (forming a small densely connected network component). By contrast, although many pairings were detected for GPCRs, individual receptors were mostly only involved in few relationships. Furthermore, the network also contained a number of isolated target pairs.

We also asked the question which functionally or therapeutically relevant information might already be available for new target pairs. Table 1 provides a summary of our database analysis. Nearly 90% of the pairs consisted of designated therapeutic targets, consistent with our approach to focus on compounds and target annotations from medicinal chemistry. However, targets from less than 20% of all pairs were implicated in the same disease and targets from less than 10% in a biological pathway relationship. For only less than 5% of all pairs, both targets were implicated in the same disease and involved in a pathway relationship. Thus, for the majority of newly identified compound-based target pairs, no therapeutic or pathway relationships were known.

**Table 1.** **Target pair information.**

**Available information**	**New target pairs (%)**
Therapeutic target	89.4
Disease relationship	19.2
KEGG pathway relationship	9.8
Disease + Pathway relationship	4.2

Reported here is the percentage of all newly identified target pairs for which disease and/or pathway relationship information is currently available.

### Exemplary relationships

**Figure 6.**
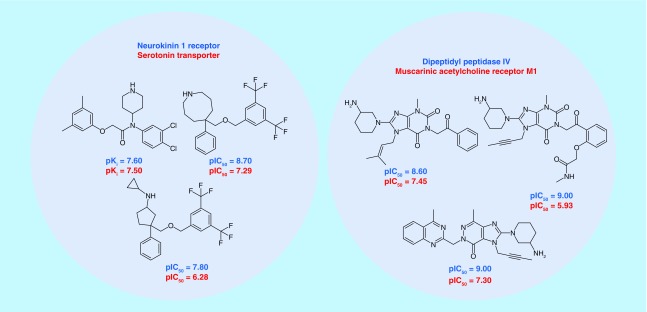
**Exemplary target pairs and compounds.** For two exemplary target pairs, active compounds, are shown (and their potency values are reported).

**Figure 7.**
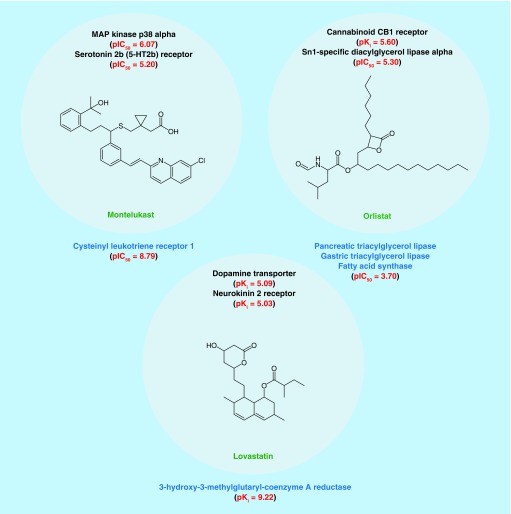
**New target hypotheses for approved drugs.** Shown here are three examples of approved drugs that participate in the formation of new target pairs, given at the top. For each drug, known target(s) from DrugBank is (are) listed at the bottom. In each case, proteins from pairs represent potential new drug targets. Potency values are provided for new target pairs and targets from DrugBank (if available in ChEMBL).

Figure 6 shows the representative examples of unexpected target relationships and corresponding active compounds such as the link between a dipeptidyl peptidase and muscarinic acetylcholine receptor M1, which shared a series of analogs. All compounds in Figure 6 had dual-target activity, in other words, they were only annotated with the targets forming the respective pairs. The examples shown also illustrate that novel target pairs involved compounds that were highly potent for one or both targets. Many such relationships can be further explored by focusing on shared active compounds and other molecules only annotated with one or the other target. In addition to target relationships, pairs also revealed new target hypotheses, as illustrated in Figure 7 for three exemplary drugs. In each case, established primary targets were taken from DrugBank. However, these drugs also participated in the formation of new target relationships on the basis of activities reported in ChEMBL. We note that the exemplary drugs shown were only weakly active against the pairs of unrelated targets. However, this might nonetheless indicate small-molecule-based relationships between these targets that merit further exploration. Borderline activities of drugs were sometimes also reported in ChEMBL for their primary targets from DrugBank, as also shown. In addition, new target relationships involving drugs might provide additional hypotheses for secondary drug targets. For example, the well-known cholesterol lowering agent lovastatin also participated in forming a relationship between a dopamine transporter and the neurokinin 2 receptor. In addition, orlistat, implicated in lipid metabolism and having several known targets, was also annotated with the cannabinoid CB1 receptor, a functionally distinct target. In addition, montelukast, an asthma and allergy medication primarily directed against the cysteinyl leukotriene receptor 1, an anti-inflammatory target, was involved in a relationships between MAPK and the 5-HT2b receptor that are critical for distinct signaling events. Thus, montelukast likely represents a polypharmacological agent. Our target pairs include nearly 150 known drugs that are involved in different relationships and whose primary and secondary targets might be compared and further investigated.

## Conclusion

In this study, we have searched for target proteins that share active compounds. The search was carried out by systematically analyzing target annotations of compounds with available high-confidence activity data taking protein sequence and family information into account. The large-scale character of our current study, considering all proteins for which medicinal chemistry efforts have been reported, sets it apart from previous attempts to search for compound relationships among groups of targets. To focus the search on distantly related or unrelated targets, intrafamily relationships were not considered. Rather, the aim of our analysis was the identification of unexpected chemical links between targets with diverse functions.

Both targets and compounds implicated in such relationships are of interest. For instance, if unrelated targets share active compounds, they have similar ligand-binding characteristics and thus might be inhibited (or activated) simultaneously if relevant compounds are administered. These insights also provide opportunities for the design of polypharmacological ligands, if targets with shared active compounds have complementary functions that are of interest for therapy, for example, if they participate in coordinated signaling and metabolic pathways. Furthermore, for drugs or other bioactive compounds involved in such relationships, new target hypotheses might be obtained.

Our current analysis has identified 286 pairs of distantly or unrelated targets that met our selection criteria, shared varying numbers of active compounds and involved a total of 141 unique proteins. Many of these pairings combined targets with distinct functions, hence providing many opportunities for follow-up investigations. For example, for implicated pairs of distantly related or unrelated targets, a search or screening effort for new active compounds might be initiated to further investigate pharmacological consequences of parallel engagement. In addition, in these instances, multitarget ligands with functional effects across different therapeutic areas might be generated and pharmacological readouts be explored.

## Future perspective

In recent years, volumes of compounds and activity data have grown in an unprecedented manner. Currently available bioactive compounds provide a rich source for information for exploring structure–activity relationships or multitarget activities. The latter topic is particularly relevant for better understanding the molecular basis of polypharmacology and for studying compound–target relationships on a large scale, as reported herein. Establishing links between distantly related or unrelated targets on the basis of active small molecules they share is of considerable interest for pharmaceutical research. Chemical links between targets were often established by large numbers of compounds, and our analysis identified more compound-based target relationships than we expected to find when planning this study. For many emerging targets, only a few active compounds are currently available, and these compounds are unlikely to be extensively tested against other targets. However, since there is no end in sight for compound data growth, we anticipate that more unexpected compound-based target relationships will become available in the future, making this an attractive area of research going forward.

As a part of our study, all relationships reported herein and the associated compound and target information are made freely available as an open access deposition [36].

**Summary points**

**Background**

- Small molecules might be specifically active against multiple target proteins.
- Different targets might share sets of active compounds.
- Sharing active compounds indicates similar ligand-binding characteristics.
- Ligand-binding features add another dimension to biological relationships.

**Materials & methods**

- Compound-based target relationships were explored on a large scale.
- Chemical relationship analysis was focused on targets from different families.
- Care was taken to base the analysis on high-confidence activity data.

**Results & discussion**

- Nearly 300 unexpected compound-based target relationships were identified.
- These relationships involved a total of 141 different proteins.
- Paired targets were often unrelated and had distinct functions.
- For a number of approved drugs, new target hypotheses were derived.

**Conclusion & future perspective**

- Bioactive compounds provide a rich source of information for connecting chemical and target space.
- Exploring compound-based target relationships is relevant for pharmaceutical research and drug repositioning.
- Further compound data growth is expected to yield more chemical links between proteins with different biological functions.
